# Anthropometric, biochemical, and nutritional risk factors for osteoporosis in Korean adults based on a large cross-sectional study

**DOI:** 10.1371/journal.pone.0261361

**Published:** 2021-12-13

**Authors:** Junghun Yoo, Bum Ju Lee

**Affiliations:** 1 KM Data Division, Korea Institute of Oriental Medicine, Daejeon, Republic of Korea; 2 Digital Health Research Division, Korea Institute of Oriental Medicine, Daejeon, Republic of Korea; Temple University School of Medicine, UNITED STATES

## Abstract

**Background:**

Osteoporosis a common bone disorder characterized by decreases in bone mass, tension, and strength. Although many previous studies worldwide have sought to identify the risk factors for osteoporosis, studies that simultaneously examine a variety of factors, such as biochemical, anthropometric and nutritional components, are very rare. Therefore, the objective of this study was to simultaneously examine the association of osteoporosis with biochemical profiles, anthropometric factors, and nutritional components in a large-scale cross-sectional study.

**Method:**

This cross-sectional study was based on data from the Korea National Health and Nutrition Examination Survey (KNHANES VI-VII) from 2015 to 2018. Based on data from 16,454 participants, logistic regression was used to examine the association between various parameters in a crude analysis and in models adjusted for confounders.

**Results:**

In men, osteoporosis was significantly associated with the anthropometric variables height and weight; the biochemical components hemoglobin, hematocrit, urea nitrogen and urine pH and creatinine; and the nutritional components total food intake, energy, water, protein, phosphorus, and kalium. However, these associations disappeared in adjusted model 2. In women, osteoporosis was significantly related to the anthropometric measures height, weight, and systolic blood pressure; the biochemical components hemoglobin, hematocrit and urine pH; and the nutritional components total food intake, water, calcium, phosphorus, and kalium. Most of these associations were maintained in the adjusted models.

**Conclusion:**

Osteoporosis was linked to various anthropometric, biochemical and urine and nutritional components in Korean women, but the association between osteoporosis and risk factors differed according to sex.

## Introduction

Osteoporosis is a major public health problem related to fragility fractures that leads to morbidity, mortality, chronic pain, disability, and low quality of life [1–3]. Due to the aging of the world population, the prevalence of osteoporosis has increased rapidly [3–7]. The US Preventive Services Task Force estimated that approximately 12.3 million elderly Americans will have osteoporosis in 2020 [1]. In the European Union, approximately 3.79 million people have osteoporotic fractures; the health care costs of osteoporotic fractures were estimated at €32 billion in 2000, and the costs are expected to double by 2050 [2]. A total of 49 million people in Australia, Europe, North America, and Japan meet the World Health Organization criteria for osteoporosis. In mainland China, the standardized prevalence of osteoporosis in elderly men and women was estimated to range from 5.04% to 7.46% in 1990 and to reach 26.28% to 39.19% by 2050 [8].

Osteoporosis-related fractures occur in one in five adult men and one in three adult women during their lifetime after the age of 50 years in Western countries [9]. Osteoporosis is associated with bone mass loss and deterioration of the microarchitecture of the bone and is caused by fragility fractures, which are related to severe pain, reduced social function, and decreased physical function [10]. For the treatment for osteoporosis and therapeutic management, rehabilitation management focuses on improving quality of life and includes strategies such as pain reduction, increased physical function, and independence of daily activities [11, 12]. For example, some studies reported that occupational training and gradual strength training after surgery [11, 13, 14] and long-term care and management of patients through a health education program at home [11, 15] were effective for managing osteoporosis. In addition, patients with postmenopausal osteoporosis were prescribed drugs such as generic bisphosphonates to reduce the risk of spine and hip fractures [9, 16], and drugs such as teriparatide and abaloparatide are known to be very effective in enhancing hip and spine bone mineral density [9, 17]. Osteoporosis is diagnosed using bone mineral density (BMD), which is determined by dual energy X-ray absorptiometry (DXA or DEXA) of the lumbar spine and hip [2–4].

Risk factors for osteoporosis are generally very diverse. Risk factors may arise from medical treatment, such as glucocorticoid treatment [5, 18, 19]; diseases and medication use [3, 4, 20–23]; biochemical factors, such as hemoglobin [20, 24], total cholesterol, high-density lipoprotein cholesterol (HDL-C), and low-density lipoprotein cholesterol LDL-C [14]; nutritional factors, such as the intake of calcium [4, 5, 7, 18, 22, 25–28] and vitamin D [4, 5, 7, 22, 26, 28–30]; sociodemographic or economic characteristics, such as age [2, 4, 5, 18, 20, 26, 30], female sex [4, 20, 26], education level [7], body size [2, 4–7, 22, 26] and body mass index (BMI) [3, 7, 18, 20, 27, 30]; menopause or premature menopause [4, 7, 22, 30]; behavior and habits, such as exercise or activity levels [3, 4, 18, 26, 30]; smoking [3–5, 18, 22, 26, 28, 31]; alcohol consumption [3, 4, 20, 26, 27, 30, 32]; and genetic factors, such as race or ethnicity [3–5, 22]. Some studies have suggested that osteoporosis is caused by a combination of these risk factors [5].

Although many previous studies worldwide have examined the risk factors for osteoporosis, studies that simultaneously investigate a variety of anthropometric, biochemical, and nutritional risk factors in the same subjects are very rare. Therefore, the objective of this study was to simultaneously examine the association of osteoporosis with biochemical profiles, anthropometric factors, and nutritional components in a large-scale cross-sectional study. Our findings and results will be useful for the treatment and prevention of osteoporosis in public health or epidemiological contexts.

## Materials and methods

### Subjects and data source

This study was based on data from the Korea National Health and Nutrition Examination Survey (KNHANES). The KNHANES is a nationally representative cross-sectional survey that has been conducted by the Korea Centers for Disease Control and Prevention (KCDC) since 2007 to examine the health and nutritional status of Koreans [33–35]. The participants are selected using a stratified, multistage probability sampling method to select household units from each survey section. The data from the Korea National Health and Nutrition Examination Survey are based on health examinations, nutrition surveys and health-related interviews with participants enrolled from 16 representative cities in the Republic of Korea. The KNHANES VI and VII collected data in 17 cities and provinces (Gyeonggi-do, Gangwon-do, Jeollabuk-do, Jeollanam-do, Chungcheongbuk-do, Chungcheongnam-do, Gyeongsangbuk-do, Gyeongsangnam-do, Jeju island, Seoul, Busan, Sejong, Daejeon, Daegu, Gwangju, Incheon, and Ulsan). Well-trained experts visited the homes of the subjects and conducted face-to-face health interviews on medical conditions using a questionnaire according to a rigorous process and protocol [33–37].

**Fig 1:**
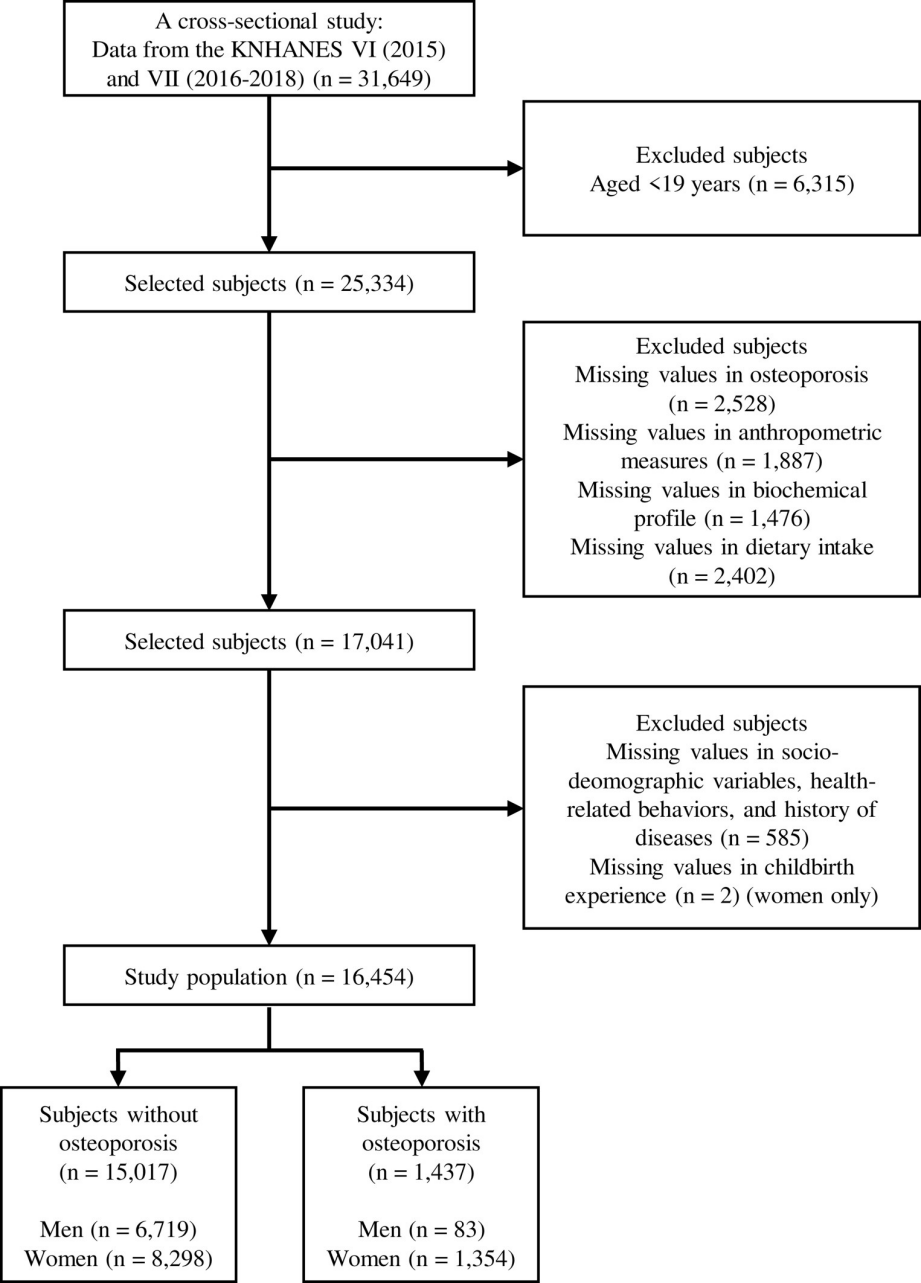
Sample selection procedure used in this study.

We used KNHANES VI and VII data from 2015 to 2018, which collected data regarding the diagnosis of osteoporosis. In KNHANES VI (2015) and VII (2016–2018), 31,649 individuals (14,452 men 17,197 women) participated. We limited our analyses to subjects older than 19 years. We excluded subjects with missing data on key covariates, including osteoporosis, covariates, blood samples, urine tests and dietary intake. The detailed sample selection procedure is shown in Fig 1.

For KNHANES VI and VII, 30 expert committees (comprising over 120 nominated experts in Korea) technically supported KNHANES for the validation and implementation of the survey. Only the survey staff members who passed intensive training course conducted the survey, and the staff members are trained frequently throughout the year to reinforce the appropriate techniques and protocol. All subjects should answer each question item about socioeconomic status and medical conditions through a self-administered questionnaire conducted by face-to-face interviews with well-trained staff members [33–37]. In addition, information on daily dietary intake based on the Korean Foods and Nutrients Database of the Rural Development Administration was collected [36]. More detailed information on the questionnaire and KNHANES survey has been provided previously [33–35].

The Institutional Review Board of the KCDC approved KNHANES VI and VII (2018-01-03-P-A), and the study was conducted in accordance with the Declaration of Helsinki. Written informed consent was obtained from the participants. This study was performed at Korea Institute of Oriental Medicine (KIOM) and approved by the Institutional Review Board of KIOM (IRB No. I-2007/006-003).

### Definition

In this study, the normal and osteoporosis groups were distinguished by whether subjects were diagnosed by a physician. Subjects with osteoporosis were identified by the question “Have you ever been diagnosed with osteoporosis by a physician?” in face-to-face interviews. All subjects answered “Yes”, “No”, or “Not applicable” based on the KCDC guidelines [33–35]. The osteoporosis group included subjects who answered “Yes”, and the normal group consisted of subjects who answered “No”. Those who answered “Not applicable” were excluded because they were adolescents or children [33–35].

### Measurements

Anthropometric measures, biochemical profiles, and dietary intake (nutritional components) were used to investigate the association between osteoporosis and risk factors. Anthropometric measures were tested according to standardized protocols [33–35]. Blood samples were collected after more than 8 h of fasting. Fasting blood glucose, glycated hemoglobin (HbA1c), total cholesterol, triglycerides, aspartate transaminase (AST), alanine transaminase (ALT), hepatitis C virus antibody, hemoglobin, hematocrit, blood urea nitrogen, and blood creatinine were measured. Urine pH, specific gravity, and creatinine were assessed. Total food intake (g), energy (kcal), water (g), protein (g), total dietary fiber (g), calcium (mg), phosphorus (mg), iron (mg), kalium (mg), vitamin A (mg), and vitamin C (mg) were assessed in our study. Detailed measurements of all variables are presented in Table 1.

**Table 1:** Measurements of the variables used in this study.

Type	Variable	Measurement method	Equipment
Anthropometric measures	Height	Measured to the nearest 0.1 cm in the standing position	Seca 225, Seca, Germany
	Weight	Measured to the nearest 0.1 kg	GL-6000-20, G-tech, South Korea
	Waist circumference	Measured to the nearest 0.1 cm at the midpoint between the iliac crest and the last rib	Seca 200, Seca, Germany
	Blood pressure and pulse rate	Assessed by averaging the values obtained for the last two of three blood pressure readings	Baumanometer Wall Unit 33 (0850), Baum, USA
Biochemical profiles	Profiles	Collected after more than 8 h of fasting	Hitachi Automatic Analyzer 7600–210, Hitachi, Japan or XN-series 9000, Sysmex, Japan or Tosoh G8, Tosoh, Japan
	Urine pH, specific gravity, and creatinine	Range of 5.0–9.0	Urisys 2400 automated urine analyzer, Roche, Germany
Dietary intake information	Total food intake, energy, water, protein, total dietary fiber, calcium, phosphorus, iron, kalium, vitamin A, and vitamin C	Estimated based on the Korean Foods and Nutrients Database of the Rural Development Administration	Obtained using the 24-h recall method

### Covariates

As potential confounders, the following socioeconomic status and demographic variables were included: age, annual income, education, occupation, marital status, drinking, smoking, BMI and childbirth experience (women only). Education level was categorized into four groups: Below elementary, middle school graduate, high school graduate, and college graduate and above. Occupation types were classified into seven groups: 1) managers and professionals; 2) clerical support workers; 3) service and sales workers; 4) skilled agricultural, forestry and fishery workers; 5) craft, plant, or machine operators and assemblers; 6) laborers; and 7) unemployed (including students and homemakers). Marital status was divided into three groups: never married, currently married, and previously married. Smoking status was defined as current smoker, previously smoked, and never smoked. Alcohol consumption over the past 12 months was classified into two groups: no and yes.

### Statistical analysis

Chi-square tests and t-tests were used to compare the prevalence of osteoporosis across covariates in the study population. To examine whether osteoporosis was related to each variable after standardization, we used univariable logistic regression in crude analysis and multiple logistic regression in adjusted models. In multiple logistic regression, models 1 and 2 each included one variable and covariates for adjustment. In men, model 1 was adjusted for age and BMI. Model 2 was adjusted for the variables in model 1 plus annual income, education, occupation, marital status, smoking, and drinking. In women, model 1 was adjusted for age and BMI. Model 2 was adjusted for the variables in model 1 plus annual income, education, occupation, marital status, smoking, drinking, and childbirth experience. Odds ratios were estimated with 95% confidence intervals. All statistical analyses were performed by using R version 3.6.2. To determine the reliability of the statistical results of the R program, we tested the results in a logistic regression with IBM SPSS version 23.

## Results

A total of 16,454 subjects (6,802 men and 9,652 women) were included in the analysis. The numbers of normal and osteoporosis subjects were 6719 (98.8%) and 83 (1.2%) men and 8298 (86.0%) and 1354 (14.0%) women, respectively.

The characteristics of the study population are shown in Table 2. The prevalence of physician-diagnosed osteoporosis was 1.2% for men and 14.0% for women. The prevalence of osteoporosis was higher among older adults; those in the lower income group; those with less than an elementary education; skilled agricultural and forestry and fishery workers; previously married adults; nondrinkers; never-smokers; and women who had experienced childbirth.

**Table 2:** Characteristics of the study population.

Variable^a^	Total (n = 16,454)		Men (n = 6,802)		Women (n = 9,652)	
	Normal group	Osteoporosis group	Normal group	Osteoporosis group	Normal group	Osteoporosis group
Total	15017 (91.3)	1437 (8.7)	6719 (98.8)	83 (1.2)***	8298 (86.0)	1354 (14.0)
Age (years)	49.3±16.1	68.2±8.6***	50.5±16.8	67.4±11.0***	48.4±15.5	68.3±8.5***
Annual income (10,000 WON)	437.9±316.9	261.4±292***	431.7±316.8	224.9±256.2***	442.9±316.9	263.6±294.0***
Education						
Below elementary	2507 (74.1)	877 (25.9)***	971 (95.9)	41 (4.1)***	1536 (64.8)	836 (35.2)***
Middle school graduate	1418 (85.5)	241 (14.5)	641 (96.8)	21 (3.2)	777 (77.9)	220 (22.1)
High school graduate	5115 (96.1)	207 (3.9)	2363 (99.6)	9 (0.4)	2752 (93.3)	198 (6.7)
College graduate or higher	5977 (98.2)	112 (1.8)	2744 (99.6)	12 (0.4)	3233 (97.0)	100 (3.0)
Occupation						
Managers and professionals	2306 (98.7)	31 (1.3)***	1119 (99.5)	6 (0.5)***	1187 (97.9)	25 (2.1)***
Clerical support workers	1726 (98.9)	20 (1.1)	838 (99.8)	2 (0.2)	888 (98.0)	18 (2.0)
Service and sales workers	1970 (94.1)	123 (5.9)	666 (99.7)	2 (0.3)	1304 (91.5)	121 (8.5)
Skilled agricultural, forestry and fishery workers	642 (84.7)	116 (15.3)	426 (97.9)	9 (2.1)	216 (66.9)	107 (33.1)
Craft, plant, or machine operators and assemblers	1549 (97.7)	36 (2.3)	1299 (99.0)	13 (1.0)	250 (91.6)	23 (8.4)
Laborers	1261 (88.1)	170 (11.9)	543 (99.1)	5 (0.9)	718 (81.3)	165 (18.7)
Unemployed (including students and homemakers)	5563 (85.5)	941 (14.5)	1828 (97.5)	46 (2.5)	3735 (80.7)	895 (19.3)
Marital status						
Currently married	10777 (92.6)	867 (7.4)***	4904 (98.6)	69 (1.4)***	5873 (88.0)	798 (12.0)***
Previously married	1575 (74)	552 (26)	375 (97.4)	10 (2.6)	1200 (68.9)	542 (31.1)
Never married	2665 (99.3)	18 (0.7)	1440 (99.7)	4 (0.3)	1225 (98.9)	14 (1.1)
Alcohol consumption						
No	3719 (82.4)	795 (17.6)***	1120 (97.5)	29 (2.5)***	2599 (77.2)	766 (22.8)***
Yes	11298 (94.6)	642 (5.4)	5599 (99.0)	54 (1.0)	5699 (90.6)	588 (9.4)
Smoking						
Current smoker	2658 (98)	53 (2)***	2248 (99.2)	19 (0.8)**	410 (92.3)	34 (7.7)***
Previously smoked	3337 (97.2)	96 (2.8)	2830 (98.3)	50 (1.7)	507 (91.7)	46 (8.3)
Never smoked	9022 (87.5)	1288 (12.5)	1641 (99.2)	14 (0.8)	7381 (85.3)	1274 (14.7)
15-second pulse count	17.7±2.2	17.5±2.2*	17.6±2.2	17.2±2.0	17.8±2.1	17.6±2.2**
Systolic blood pressure (mmHg)	118±16.5	126.2±17.7***	121.0±14.9	127.1±15.9**	115.5±17.3	126.1±17.8***
Diastolic blood pressure (mmHg)	75.7±10.1	73.7±9.4***	78.0±10.2	74.8±9.1**	73.8±9.6	73.6±9.4
Height (cm)	163.6±9	153.4±6.8***	170.4±6.8	165.3±7.9***	158.0±6.3	152.7±6.0***
Weight (kg)	64.2±12.4	56.3±8.8***	71.3±11.9	64.5±12.5***	58.5±9.4	55.8±8.3***
Waist circumference (cm)	82.2±10.2	82.5±9.2	86.6±9.1	86.0±9.7	78.7±9.7	82.3±9.2***
BMI	23.9±3.6	23.9±3.3	24.5±3.4	23.6±3.7*	23.5±3.6	23.9±3.3***
Childbirth experience						
Yes					6800 (83.9)	1305 (16.1)***
No					184 (89.3)	22 (10.7)
Have never been pregnant					1314 (98.0)	27 (2.0)

^a^ Variables are given as the mean±standard deviation or number (%).

P-values were obtained by t-test or chi-square test.

*P < 0.05

**P < 0.01

***P < 0.001.

Table 3 presents the association of osteoporosis with anthropometric, biochemical, and dietary factors in men. Among the anthropometric measures, the osteoporosis group was older than the normal group (OR = 1.08 [1.06–1.10]) and had a lower BMI (OR = 0.91 [0.85–0.98]). The osteoporosis group was more likely to be shorter (OR = 0.91 [0.88–0.93] in the crude model, adj. OR = 0.96 [0.93–1.00] in model 1) and weigh less (OR = 0.94 [0.92–0.96] in the crude model, adj. OR = 0.95 [0.91–1.00] in model 1). The association of systolic blood pressure (SBP) (OR = 1.02 [1.01–1.04]) and diastolic blood pressure (OR = 0.97 [0.95–0.99]) with osteoporosis was statistically significant in the crude model, but the statistical significance disappeared in models 1 and 2.

**Table 3:** Association of osteoporosis with anthropometric measures, biochemical profile, and dietary intake in men.

Variables		Normal group	Osteoporosis group	Crude	Model 1	Model 2
		Mean±SD	Mean±SD	OR (95% CI)	OR (95% CI)	OR (95% CI)
Anthropometric measures	Age	50.49±16.84	67.42±10.97	1.08 (1.06–1.10)***		
	BMI	24.50±3.374	23.55±3.690	0.91 (0.85–0.98)*		
	Height (cm)	170.4±6.802	165.3±7.851	0.91 (0.88–0.93)***	0.96 (0.93–1.00)*	0.98 (0.94–1.02)
	Weight (kg)	71.33±11.91	64.50±12.52	0.94 (0.92–0.96)***	0.95 (0.91–1.00)*	0.97 (0.93–1.02)
	Waist circumference (cm)	86.59±9.087	85.97±9.685	0.99 (0.97–1.02)	1.01 (0.95–1.06)	1.01 (0.95–1.06)
	15-second pulse count	17.55±2.240	17.17±2.029	0.92 (0.82–1.02)	0.93 (0.83–1.03)	0.90 (0.80–1.00)
	Systolic blood pressure	121.0±14.88	127.1±15.87	1.02 (1.01–1.04)***	1.01 (0.99–1.02)	1.01 (0.99–1.02)
	Diastolic blood pressure	78.01±10.17	74.82±9.105	0.97 (0.95–0.99)**	1.00 (0.98–1.03)	1.01 (0.98–1.03)
Biochemical profile	Fasting blood glucose (mg/L)	104.2±26.25	107.0±29.18	1.09 (0.89–1.29)	0.94 (0.73–1.15)	0.94 (0.73–1.14)
	Glycated hemoglobin (%)	5.759±0.911	5.889±0.816	1.13 (0.92–1.33)	0.91 (0.71–1.13)	0.90 (0.71–1.11)
	Total cholesterol (mg/dL)	190.2±37.50	182.2±34.97	0.80 (0.64–1.00)	0.98 (0.79–1.22)	1.00 (0.81–1.24)
	Triglycerides (mg/dL)	160.2±132.4	155.2±134.1	0.96 (0.73–1.17)	1.12 (0.86–1.34)	1.13 (0.86–1.36)
	Aspartate transaminase (IU/L)	25.28±17.66	25.41±10.90	1.01 (0.73–1.12)	1.01 (0.72–1.13)	0.99 (0.68–1.14)
	Alanine transaminase (IU/L)	27.54±21.18	23.00±15.91	0.70 (0.48–0.95)*	0.99 (0.68–1.29)	0.96 (0.66–1.26)
	Hepatitis C virus antibody	0.136±0.894	0.116±0.361	0.97 (0.57–1.14)	0.91 (0.49–1.10)	0.90 (0.47–1.08)
	Hemoglobin (g/dL)	15.28±1.267	14.53±1.281	0.63 (0.54–0.75)***	0.89 (0.73–1.10)	0.90 (0.74–1.10)
	Hematocrit (%)	46.03±3.660	44.04±3.739	0.64 (0.55–0.77)***	0.91 (0.74–1.12)	0.91 (0.74–1.12)
	Blood urea nitrogen (mg/dL)	15.35±4.869	16.99±5.434	1.18 (1.03–1.32)**	1.00 (0.82–1.17)	1.02 (0.84–1.17)
	Blood creatinine (mg/dL)	0.966±0.302	0.971±0.192	1.01 (0.74–1.12)	0.88 (0.62–1.09)	0.90 (0.64–1.09)
	Urine pH	5.765±0.791	5.970±0.951	1.27 (1.03–1.55)*	1.13 (0.92–1.36)	1.15 (0.94–1.39)
	Urine specific gravity	1.020±0.006	1.019±0.006	0.82 (0.66–1.01)	1.06 (0.83–1.35)	1.03 (0.81–1.31)
	Urine creatinine (mg/dL)	177.4±88.74	139.9±70.28	0.59 (0.44–0.76)***	0.91 (0.67–1.21)	0.87 (0.64–1.15)
Dietary intake (nutritional components)	Total food intake (g)	1823±919.4	1406±706.0	0.53 (0.39–0.70)***	0.76 (0.56–1.01)	0.92 (0.68–1.21)
	Energy (kcal)	2362±1015	1982±830.6	0.61 (0.45–0.80)***	0.88 (0.65–1.17)	1.01 (0.74–1.33)
	Water (g)	1147±703.8	887.1±568.1	0.60 (0.44–0.80)***	0.78 (0.58–1.03)	0.94 (0.70–1.21)
	Protein (g)	84.39±52.23	66.19±34.25	0.50 (0.35–0.71)***	0.84 (0.57–1.12)	1.01 (0.70–1.20)
	Total dietary fiber (g)	27.59±14.59	25.61±13.12	0.86 (0.67–1.08)	0.85 (0.66–1.06)	0.95 (0.74–1.18)
	Calcium (mg)	572.8±341.0	514.1±428.9	0.81 (0.62–1.04)	0.98 (0.76–1.22)	1.08 (0.85–1.31)
	Phosphorus (mg)	1238±619.6	1044±501.2	0.63 (0.46–0.83)**	0.87 (0.64–1.13)	1.04 (0.77–1.23)
	Iron (mg)	15.20±10.10	13.67±7.224	0.80 (0.59–1.05)	0.92 (0.67–1.15)	1.00 (0.75–1.18)
	Kalium (mg)	3242±1548	2843±1324	0.73 (0.56–0.94)*	0.86 (0.66–1.10)	0.99 (0.76–1.24)
	Vitamin A (μg RE)	711.8±794.7	620.5±622.6	0.84 (0.59–1.09)	0.97 (0.72–1.17)	1.01 (0.79–1.19)
	Vitamin C (mg)	74.16±89.62	60.93±65.83	0.80 (0.56–1.05)	0.86 (0.61–1.11)	0.96 (0.69–1.21)

The results (odds ratio and p-value) were obtained by binary logistic regression.

*P < 0.05

**P < 0.01

***P < 0.001.

Model 1: adjusted for age and BMI.

Model 2: adjusted for age, BMI, annual income, education, occupation, marital status, smoking, and drinking.

Continuous variables are represented as the mean ± standard deviation.

OR: odds ratio, CI: confidence interval, SD: standard deviation.

Regarding biochemical factors, osteoporosis was statistically significantly associated with alanine transaminase levels (OR = 0.70 [0.48–0.96]), hemoglobin levels (OR = 0.63 [0.54–0.75]), urine pH (OR = 1.27 [1.03–1.55]), and urine creatinine levels (OR = 0.59 [0.44–0.76]) in the crude model, but these associations were not statistically significant in models 1 and 2.

Among dietary factors, the osteoporosis group was more likely to have a lower total food intake (OR = 0.53 [0.39–0.70]) and lower intake of energy (OR = 0.61 [0.45–0.80]), water (OR = 0.60 [0.44–0.80]), protein (OR = 0.50 [0.35, 0.71], phosphorus (OR = 0.63 [0.46–0.83]) and kalium (OR = 0.73 [0.56–0.94]) in the crude model. However, the associations of dietary factors with osteoporosis became nonsignificant in model 1 and model 2.

Table 4 shows the association of osteoporosis with anthropometric, biochemical, and dietary factors in women. Age (OR = 1.11 [1.11–1.12]) and BMI (OR = 1.04 [1.02–1.05]) were statistically significantly related to osteoporosis. In all models, the osteoporosis group was more likely to be shorter (OR = 0.87 [0.86–0.88] in the crude model, adj. OR = 0.98 [0.97–0.99] in model 1, adj. OR = 0.99 [0.97–1.00] in model 2) and weigh less (OR = 0.97 [0.96–0.97] in the crude model, adj. OR = 0.97 [0.96–0.99] in model 1, adj. OR = 0.98 [0.96–1.00] in model 2). SBP was associated with osteoporosis (adj. OR = 0.99 [0.99–1.00] in model 1 and 2).

**Table 4:** Association of osteoporosis with anthropometric measures, the biochemical profile, and dietary intake in women.

Variables		Normal group	Osteoporosis group	Crude	Model 1	Model 2
		Mean±SD	Mean±SD	OR (95% CI)	OR (95% CI)	OR (95% CI)
Anthropometric measures	Age	48.40±15.50	68.30±8.465	1.11 (1.11–1.12)***		
	BMI	23.45±3.632	23.95±3.267	1.04 (1.02–1.05)***		
	Height (cm)	158.0±6.295	152.7±5.985	0.87 (0.86–0.88)***	0.98 (0.97–0.99)**	0.99 (0.97–1.00)*
	Weight (kg)	58.51±9.433	55.82±8.306	0.97 (0.96–0.97)***	0.97 (0.96–0.99)**	0.98 (0.96–1.00)*
	Waist circumference (cm)	78.69±9.749	82.30±9.157	1.04 (1.03–1.04)***	1.01 (0.99–1.02)	1.01 (0.99–1.02)
	15-second pulse count	17.75±2.147	17.55±2.234	0.96 (0.93–0.98)**	1.00 (0.97–1.03)	0.99 (0.96–1.02)
	Systolic blood pressure	115.5±17.33	126.1±17.76	1.03 (1.03–1.03)***	0.99 (0.99–1.00)**	0.99 (0.99–1.00)***
	Diastolic blood pressure	73.79±9.610	73.63±9.445	1.00 (0.99–1.00)	1.00 (1.00–1.01)	1.00 (1.00–1.01)
Biochemical profile	Fasting blood glucose (mg/L)	97.59±20.82	103.3±24.55	1.23 (1.17–1.29)***	0.95 (0.89–1.01)	0.94 (0.88–1.01)
	Glycated hemoglobin (%)	5.624±0.722	5.933±0.847	1.36 (1.30–1.43)***	0.96 (0.90–1.02)	0.95 (0.89–1.01)
	Total cholesterol (mg/dL)	193.9±36.89	193.0±39.41	0.98 (0.92–1.03)	0.99 (0.93–1.05)	0.99 (0.93–1.05)
	Triglycerides (mg/dL)	114.0±82.82	128.8±89.07	1.15 (1.10–1.22)***	0.97 (0.91–1.04)	0.96 (0.89–1.03)
	Aspartate transaminase (IU/L)	20.96±10.12	23.77±9.822	1.25 (1.18–1.31)***	1.07 (1.01–1.13)*	1.06 (0.99–1.12)
	Alanine transaminase (IU/L)	17.94±13.58	19.79±12.13	1.12 (1.07–1.17)***	1.07 (1.00–1.14)*	1.06 (0.99–1.13)
	Hepatitis C virus antibody	0.109±0.666	0.185±1.064	1.07 (1.03–1.12)**	1.01 (0.96–1.06)	1.00 (0.95–1.05)
	Hemoglobin (g/dL)	13.12±1.161	13.14±1.082	1.02 (0.96–1.08)	1.09 (1.02–1.17)*	1.08 (1.01–1.16)*
	Hematocrit (%)	40.37±3.166	40.34±3.207	0.99 (0.94–1.05)	1.09 (1.02–1.16)*	1.09 (1.02–1.16)*
	Blood urea nitrogen (mg/dL)	13.75±4.390	16.42±5.051	1.65 (1.57–1.74)***	1.05 (0.98–1.11)	1.04 (0.98–1.11)
	Blood creatinine (mg/dL)	0.706±0.191	0.735±0.262	1.12 (1.06–1.20)***	0.93 (0.86–0.99)	0.95 (0.88–1.00)
	Urine pH	5.817±0.828	6.003±0.911	1.24 (1.17–1.31)***	1.14 (1.07–1.22)***	1.14 (1.07–1.21)***
	Urine specific gravity	1.018±0.007	1.017±0.005	0.76 (0.71–0.80)***	0.97 (0.90–1.05)	0.96 (0.89–1.03)
	Urine creatinine (mg/dL)	135.1±77.66	101.1±53.38	0.57 (0.53–0.61)***	0.92 (0.84–1.00)	0.92 (0.84–1.00)
Dietary intake (nutritional components)	Total food intake (g)	1428±695.5	1230±680.5	0.72 (0.67–0.77)***	1.05 (0.98–1.13)	1.10 (1.02–1.18)*
	Energy (kcal)	1717±728.0	1530±636.0	0.74 (0.69–0.79)***	1.03 (0.96–1.11)	1.06 (0.98–1.14)
	Water (g)	962.6±556.5	831.3±561.8	0.76 (0.72–0.82)***	1.05 (0.98–1.13)	1.10 (1.02–1.18)**
	Protein (g)	61.07±31.95	50.46±26.06	0.64 (0.60–0.69)***	1.03 (0.95–1.11)	1.07 (0.99–1.16)
	Total dietary fiber (g)	23.66±13.90	24.31±14.33	1.05 (0.99–1.11)	1.06 (0.99–1.12)	1.08 (1.01–1.15)*
	Calcium (mg)	467.4±300.0	427.8±289.4	0.86 (0.80–0.91)***	1.08 (1.01–1.15)*	1.10 (1.03–1.18)**
	Phosphorus (mg)	947.8±433.6	839.7±409.7	0.75 (0.70–0.80)***	1.06 (0.99–1.13)	1.10 (1.02–1.18)*
	Iron (mg)	11.88±7.222	11.39±8.653	0.93 (0.87–0.99)*	1.06 (0.99–1.13)	1.06 (1.00–1.13)
	Kalium (mg)	2669±1375	2520±1429	0.89 (0.84–0.95)***	1.05 (0.98–1.12)	1.07 (1.01–1.15)*
	Vitamin A (μg RE)	602.6±750.3	527.0±585.9	0.85 (0.78–0.92)***	1.02 (0.94–1.09)	1.03 (0.96–1.11)
	Vitamin C (mg)	72.66±101.0	71.34±89.56	0.99 (0.92–1.04)	1.05 (0.99–1.11)	1.06 (1.00–1.13)*

The results (odds ratio and p-value) were obtained by binary logistic regression.

*P < 0.05

**P < 0.01

***P < 0.001.

Model 1: adjusted for age and BMI.

Model 2: adjusted for age, BMI, annual income, education, occupation, marital status, smoking, drinking, and childbirth experience.

Continuous variables are represented as the mean ± standard deviation.

OR: odds ratio, CI: confidence interval, SD: standard deviation.

Regarding the biochemical factors, women with osteoporosis tended to have higher aspartate transaminase levels (OR = 1.25 [1.18–1.31] in the crude model, adj. OR = 1.07 [1.01–1.13] in model 1) and alanine transaminase levels (OR = 1.12 [1.07–1.17] in the crude model, adj. OR = 1.07 [1.00–1.14] in model 1) than women without osteoporosis. These associations were not statistically significant in model 2. Additionally, women in the osteoporosis group were more likely to have higher hemoglobin levels (adj. OR = 1.09 [1.02–1.17] in model 1, adj. OR = 1.08 [1.01–1.16] in model 2) and hematocrit levels (adj. OR = 1.09 [1.02–1.16] in model 1, adj. OR = 1.09 [1.02–1.16] in model 2). Urine pH was statistically significantly associated with osteoporosis in all models (OR = 1.24 [1.17–1.31] in the crude model, adj. OR = 1.14 [1.07–1.22] in model 1, adj. OR = 1.14 [1.07–1.21] in model 2).

Regarding dietary intake, osteoporosis was significantly associated with total food intake (adj. OR = 1.10 [1.02–1.18] in model 2) and with the intake of water (adj. OR = 1.10 [1.02–1.18] in model 2), total dietary fiber (adj. OR = 1.08 [1.01–1.15] in model 2), calcium (adj. OR = 1.10 [1.03–1.18] in model 2), phosphorus (adj. OR = 1.10 [1.02–1.18] in model 2), kalium (adj. OR = 1.07 [1.01–1.15] in model 2), and vitamin C (adj. OR = 1.06 [1.00–1.13] in model 2).

## Discussion

Osteoporosis is a skeletal disorder that results in an increase in fractures of the spine, hip, and other bones [5], and osteoporosis and fragility fractures are among the most important causes of disability [1–3]. In summary, the risk factors for osteoporosis are known to arise from various risk factors, including biochemical profile [20, 23, 24], sociodemographic or economic characteristics [2–7, 18, 20, 22, 26, 30], behavior or activity [3–5, 18, 20, 22, 26–28, 30–32], nutritional intake [4, 5, 7, 18, 22, 25–30], genetics [3–5, 22], and disease, treatments or medications [3–5, 18–23].

Previous studies have presented conflicting arguments regarding whether biochemical factors are associated with osteoporosis. For example, Lian et al. [23] reported that hypertension, coronary heart disease, hyperlipidemia, diabetes, and smoking were significantly related to osteoporosis in elderly men and women in China. They argued that total cholesterol and LDL-C were risk factors for osteoporosis and that HDL-C and weight were protective factors against osteoporosis. Asaoka et al. [20] investigated the association of osteoporosis with several sociodemographic characteristics and Helicobacter pylori positivity in Japanese men and women and suggested that increasing age, female sex, lower alcohol consumption, lower BMI, lower hemoglobin, and Helicobacter pylori positivity were risk factors for osteoporosis, but diabetes and hypertension were not associated with osteoporosis. Yoon et al. [24] examined the association of BMD in the lumbar spine and femoral neck with red blood cells, hemoglobin, and hematocrit in Korean university students and argued that lower hemoglobin levels were linked to lower BMD and that hemoglobin levels were the best indicator of abnormal BMD. In contrast, Sánchez-Rodríguez et al. [38] reported that glucose, urea, creatinine, urate, cholesterol, triglycerides, HDL-C, albumin, hemoglobin, and hematocrit were not associated with osteoporosis in elderly Mexican adults. Our findings were consistent with the results of previous studies indicating that hemoglobin was associated with osteoporosis [20, 24] and that cholesterol, glucose, creatinine, and triglycerides were not [38]. Our results showed that hemoglobin levels were related to osteoporosis in women, but cholesterol, glucose, creatinine, and triglycerides were not associated with osteoporosis in either men or women in adjusted models.

Regarding nutritional components, for example, Woo et al. [39] argued that SBP and dietary calcium intake were associated with bone mineral density and osteoporosis in elderly men and women in Hong Kong. Ilesanmi-Oyelere and Kruger [40] suggested that phosphorus, calcium, fiber, potassium, magnesium, and vitamin D and K were important for BMD based on a literature review on postmenopausal osteoporosis. Another study by Ilesanmi-Oyelere et al. [41] argued that calcium, niacin, protein, and riboflavin intake was associated with spine BMD and high phosphorus, calcium, and riboflavin intake was positively associated with femoral neck and spine BMD. Collier et al. [30] argued that vitamin D deficiency was associated with bone turnover, bone loss, and osteoporosis. Kim et al. [42] documented that dietary food intake was associated with osteoporosis in elderly Koreans, and vitamin B2 or vitamin C was related to the disease. Kim et al. [43] argued that the intake of vitamin C, sodium, and zinc was positively related to bone mass or T-scores in postmenopausal Korean women. Lane [5] reported that risk factors for osteoporosis were smoking, small body size, low bone mass, low physical activity, ethnicity, history of fractures, and low intake of vitamin D and calcium. Holm et al. [18] documented that high BMI, old age, smoking, high amounts of exercise, hyperthyroidism, use of thiazide diuretics, previous osteoporosis treatment and osteoporotic fracture, and calcium supplementation intake were indicators of osteoporosis in women. Aloia et al. [28] reported that smoking was associated with a high risk of osteoporosis, and sufficient intake of vitamin D and calcium was associated with a low risk of osteoporosis in postmenopausal women. Furthermore, Gimigliano et al. [44] examined associations among vitamin D, fat mass, and skeletal muscle and the effects of the combination of obesity and hypovitaminosis D in postmenopausal women. They reported that hypovitaminosis D was related to impaired muscle function and that the combination of hypovitaminosis D and overweight elicited a negative effect on muscle mass and function. Also, Ringe [45] argued that alfacalcidol had shown efficacy in the treatment and prevention of osteoporosis and fractures related to osteoporosis both in monotherapy and combined therapy with other osteoporotic drugs in postmenopausal women and men with osteoporosis. Keramat et al. [7] argued that lower education level, BMI, age at menopause, age at menarche, calcium supplementation, and history of fracture were significantly associated with the risk of osteoporosis in postmenopausal women in Iran and India. They documented that high weight and BMI were protective factors, and short stature was a risk factor for osteoporosis. Our findings agree with the results of previous studies [5, 7, 18, 28, 40, 41] indicating that calcium and/or phosphorus were related to osteoporosis in women. However, our results were different from those of a previous study [39]. Our results indicated that osteoporosis was associated with calcium only in women and not in men.

In addition to anthropometric factors and sociodemographic and economic characteristics, numerous studies have argued that many variables related to obesity and sociodemographic characteristics are associated with osteoporosis. For example, in a systematic review of 34 guidelines based on the Appraisal of Guidelines for Research and Evaluation instrument in terms of exercise, physical activity, or rehabilitation for osteoporosis therapy, Iolascon et al. [46] reported that therapeutic exercise or rehabilitation at moderate to high intensity was recommended for the management of subjects with fragility fractures and osteoporosis. Siris et al. [3] documented that aging, cigarette smoking, history of fracture, use of cortisone, and Hispanic or Asian ethnicity were related to a high risk of osteoporosis and that exercise, alcohol consumption, estrogen use, higher BMI, and African American ethnicity were associated with a low risk of osteoporosis. Collier et al. [30] mentioned that high alcohol consumption, low BMI, physical inactivity, previous fracture, and premature menopause were major independent risk factors for osteoporosis or osteoporotic fractures. Pouresmaeili et al. [26] documented that osteoporosis risk factors included weight loss, smoking, alcohol consumption, stress, insufficient physical activity and nutrition intake, aging, sex, and family history. Heidari et al. [47] reported that osteoporosis was associated with obesity and education level in postmenopausal women. Reginster and Burlet [2] argued that the strongest predictor of osteoporosis is older age and indicated that a history of hip fracture, low weight, and several diseases were associated with fractures. Assantachai et al. [6] reported that risk factors for osteoporosis in elderly Thai people were mental health, height, and lean body mass. Seeman et al. [27] reported that smoking, alcohol consumption, calcium levels and bone metabolism were associated with a high risk of osteoporosis, and obesity was protective against osteoporosis in men. Kanis [4] and Kanis and McCloskey [22] reported that female sex, age, premature menopause, White and Asian ethnicity, low bone mineral density, low weight, smoking, excessive drinking, inactivity, low calcium intake and insufficient vitamin D intake were risk factors for osteoporosis. Mangiafico et al. [48] reported that central aortic SBP was higher among postmenopausal women with osteoporosis than in those without osteoporosis. Our findings were consistent with those of previous studies indicating that BMI or obesity was linked to osteoporosis in women [2–4, 6, 22, 26, 30, 47] and men [6, 8].

Our study has several limitations. First, cause-effect relationships cannot be explained given the nature of cross-sectional studies. Second, we cannot guarantee that the findings of this study are comparable to those of studies conducted in other countries due to differences in socioeconomic characteristics and dietary and obesity criteria among ethnic groups. Third, our results may be affected by a number of biases including survey bias and self-reported diagnosis of osteoporosis. Fourth, our study did not consider the presence and assessment of fragility fractures in the osteoporosis subject group or the assessment of some covariates including physical activity or serum vitamin D levels. Finally, compared to the number of original samples in KNHANES, a large number of samples was excluded from the analysis.

In conclusion, we examined the association between osteoporosis and biochemical, anthropometric and nutritional components in a large-scale cross-sectional study. We found various risk factors for osteoporosis among anthropometric measures, blood and urine factors and nutritional components in Korean women. We hope that our findings and results will provide useful information for the treatment or prevention of osteoporosis in public health or epidemiology contexts.
